# Exercise Training Stabilizes RyR2-Dependent Ca^2+^ Release in Post-infarction Heart Failure

**DOI:** 10.3389/fcvm.2020.623922

**Published:** 2021-01-25

**Authors:** Tore Kristian Danielsen, Mani Sadredini, Ravinea Manotheepan, Jan Magnus Aronsen, Michael Frisk, Marie Haugsten Hansen, Kjetil Wessel Andressen, Karina Hougen, Finn Olav Levy, William E. Louch, Ole Mathias Sejersted, Ivar Sjaastad, Mathis Korseberg Stokke

**Affiliations:** ^1^Institute for Experimental Medical Research, Oslo University Hospital, University of Oslo, Oslo, Norway; ^2^Kristian Gerhard (KG) Jebsen Centre for Cardiac Research, University of Oslo, Oslo, Norway; ^3^Bjørknes College, Oslo, Norway; ^4^Department of Pharmacology, Institute of Clinical Medicine, Oslo University Hospital, University of Oslo, Oslo, Norway

**Keywords:** exercise training, arrhythmias, cardiac ryanodine receptor, heart failure, myocardial infarction

## Abstract

**Aim:** Dysfunction of the cardiac ryanodine receptor (RyR2) is an almost ubiquitous finding in animal models of heart failure (HF) and results in abnormal Ca^2+^ release in cardiomyocytes that contributes to contractile impairment and arrhythmias. We tested whether exercise training (ET), as recommended by current guidelines, had the potential to stabilize RyR2-dependent Ca^2+^ release in rats with post-myocardial infarction HF.

**Materials and Methods:** We subjected male Wistar rats to left coronary artery ligation or sham operations. After 1 week, animals were characterized by echocardiography and randomized to high-intensity interval ET on treadmills or to sedentary behavior (SED). Running speed was adjusted based on a weekly VO_2max_ test. We repeated echocardiography after 5 weeks of ET and harvested left ventricular cardiomyocytes for analysis of RyR2-dependent systolic and spontaneous Ca^2+^ release. Phosphoproteins were analyzed by Western blotting, and beta-adrenoceptor density was quantified by radioligand binding.

**Results:** ET increased VO_2max_ in HF-ET rats to 127% of HF-SED (*P* < 0.05). This coincided with attenuated spontaneous SR Ca^2+^ release in left ventricular cardiomyocytes from HF-ET but also reduced Ca^2+^ transient amplitude and slowed Ca^2+^ reuptake during adrenoceptor activation. However, ventricular diameter and fractional shortening were unaffected by ET. Analysis of Ca^2+^ homeostasis and major proteins involved in the regulation of SR Ca^2+^ release and reuptake could not explain the attenuated spontaneous SR Ca^2+^ release or reduced Ca^2+^ transient amplitude. Importantly, measurements of beta-adrenoceptors showed a normalization of beta_1_-adrenoceptor density and beta_1_:beta_2_-adrenoceptor ratio in HF-ET.

**Conclusion:** ET increased aerobic capacity in post-myocardial infarction HF rats and stabilized RyR2-dependent Ca^2+^ release. Our data show that these effects of ET can be gained without major alterations in SR Ca^2+^ regulatory proteins and indicate that future studies should include upstream parts of the sympathetic signaling pathway.

## Introduction

Current guidelines recommend exercise training (ET) as part of rehabilitation programs after myocardial infarction (MI) and for patients with heart failure (HF) (1, 2). ET has a range of beneficial cardiovascular effects (3) including improved aerobic capacity and cardiac contractile function (4–6) and decreases mortality after MI and in patients with HF (7, 8). However, approximately half of all patients with HF still die of ventricular arrhythmias (9). To exploit fully the therapeutic potential of ET in HF, we need a better understanding of its effects on the mechanisms for arrhythmias in HF.

The mechanism that underlies increased risk of arrhythmias in post-MI HF is multifactorial and includes fibrosis, altered expression or function of ion channels, and perturbed Ca^2+^ homeostasis (10). An almost ubiquitous finding in animal models of post-MI HF is a dysfunction of the cardiac ryanodine receptor (RyR2), i.e., the Ca^2+^ release channel of the sarcoplasmic reticulum (SR) (11, 12). This channel is essential in normal cellular Ca^2+^ handling and excitation–contraction coupling (13). RyR2 dysfunction can lead to diastolic Ca^2+^ leak from the SR, which contributes to contractile impairment and arrhythmias (14). Inhibition of SR Ca^2+^ leak has been found to prevent arrhythmias in animal models of HF, as well as arrhythmogenic events in cardiac tissue taken from patients with HF, and is a potential future therapeutic strategy (15, 16). However, no drugs that specifically target RyR2 are clinically available. Based on results from mouse models that show RyR2 dysfunction, ET might have the potential to stabilize RyR2 function and prevent arrhythmias (17, 18). However, there is a scarcity of data that support such an effect of ET in post-MI HF, and therefore, there is a need for rigorous experimental data from clinically relevant models.

## Materials and Methods

### Ethical Approval

This investigation was approved by the Norwegian National Committee for Animal Welfare under the Norwegian Animal Welfare Act (FOTS ID: 4173 and 6577). It conformed to the National Institutes of Health guidelines (NIH Publication No. 85-23, revised 1996, US).

### Animal Model of Post-infarction Heart Failure

A total of 52 male Wistar rats were included in the study. They either underwent sham operations or were subjected to MI by left coronary artery ligation. Left coronary artery ligation was performed through a thoracotomy under general anesthesia, which was achieved by inhalation of 65% N_2_O, 32% O_2_, and 2.5% isoflurane through an endotracheal tube, as previously described (19). Through a 2-cm incision in the skin over the sternum, the cutis on the left thorax was loosened from the underlying layer, and a left thoracotomy was performed in the fourth intercostal space. The pericardial sac was opened, and the left coronary artery was ligated ~1 mm beneath the left atrium. In the control (sham) rats, the same operating procedure was employed, but the left coronary artery ligation was not performed. The skin was closed by sutures. Buprenorphine 0.2 mg/kg was administered subcutaneously for analgesia.

Rats with MI were evaluated after 1 week by 2D M-mode echocardiography. Based on previously established criteria, rats that exhibited a left atrial diameter of >5.0 mm were included in the HF group (15, 17). Rats within the same group (sham or HF) were paired according to weight and were randomly assigned either to a 5-week high-intensity ET program or to a sedentary, i.e. non-exercising, control group. The rats were housed together in cages under a 12:12 h light:dark cycle, with free access to water and food.

### Protocol for Exercise Training

ET was initiated 1 week after coronary artery ligation. Separate treadmill and metabolic chambers were used for exercise training and for weekly measurements of maximal oxygen uptake (VO_2max_) (Columbus Instruments, OH, USA), respectively. Three days before the first VO_2max_-test, rats were habituated to the treadmill with daily 15-min exercise bouts at walking pace.

High-intensity exercise training has been shown to increase aerobic capacity in rats more than moderate exercise training (6). Pilot data, as well as previous publications, showed that the effect on VO_2max_ reached a plateau at 5 weeks (6, 20). Therefore, all ET rats completed a 5-week high-intensity ET protocol of treadmill running. Each training session lasted for 1 h. It consisted of a 10-min warm-up period and five 8-min intervals at 80–90% of the running speed that had been found to produce VO_2max_, interspersed by 2-min rest periods at 60% running speed. The treadmill was set at an inclination of 25° at all times. The running speed was individualized for all rats according to a weekly VO_2max_-test to keep the intensity constant throughout the ET program. SED rats ran on the treadmill at 60% of the speed that was found to produce VO_2max_ for 15 min, 2 days per week. All rats in the ET groups continued daily exercise until 1 day before euthanasia.

### Echocardiography

Echocardiographic parameters were recorded with a Vevo2100 System (Fujifilm VisualSonics Inc., Canada) in rats that were anesthetized by a mixture of 65% N_2_O, 33% O_2_, and 2% isoflurane by mask ventilation. Echocardiography was performed 1 week after coronary ligation to enable stratification, and it was repeated at completion of the ET program (21).

### Cardiomyocyte Calcium Imaging

Ventricular cardiomyocytes were isolated using constant-pressure perfusion of the coronary arteries with collagenase-containing solution (solution containing in mM 130 NaCl, 25 HEPES, 22 D-glucose, 5.4 KCl, 0.5 MgCl_2_, 0.4 NaH_2_PO_4_, adjusted to pH 7.4 with NaOH), as previously described (22). The hearts were mounted on a Langendorff setup and were perfused retrogradely through the aorta with a 37°C solution that contained 0.08 mM Ca^2+^ and 200 U/ml collagenase type 2 (Worthington Biochemical Corporation, Lakewood, NJ, USA). The hearts were perfused for 20 min before the left ventricular tissue was excised rapidly, and the infarcted area was removed. The ventricular tissue was then cut into small pieces and was gently mixed with a cutoff Pasteur pipette for about 1 min in a buffer that contained 1% bovine serum albumin (BSA) (Sigma Aldrich) and 0.02 U/ml deoxyribonuclease 1 (Worthington Biochemical Corporation, Lakewood, NJ, USA). The solution with the ventricular tissue was then filtered through a nylon mesh (pore diameter, 200 μm). After sedimentation, the cells were resuspended three times in 1% BSA solutions with increasing Ca^2+^ concentration (0.1, 0.2, and 0.5 mM, respectively). The cells were used for experiments within 10 h.

Whole-cell Ca^2+^ imaging was performed with a Zeiss Axiovert 200M microscope (Carl Zeiss Microscopy, LCC, NY, USA). Left-ventricular cardiomyocytes were field stimulated at 1, 2, and 4 Hz by a 3-ms symmetrical bipolar pulse, which was ~20% above the voltage threshold for contraction of the individual cardiomyocytes. The experimental superfusate was based on modified HEPES–Tyrode's solution, and contained (in mM) HEPES 5, NaCl 140, KCl 5.4, MgCl_2_ 0.5, glucose 5.5, NaH_2_PO_4_ 0.4, and CaCl_2_ 1. pH was adjusted to 7.4 with NaOH. Cytosolic Ca^2+^ was visualized the by use of 5 μM fluo-4 acetomethyl (AM) ester (Molecular Probes, Eugene, Oregon, USA), with 10-min loading before experiments. All experiments were performed at 37°C. For beta-adrenoceptor activation, 10 nM isoprenaline sulfate (ISO) (NAF, Norway) was added to the modified HEPES–Tyrode's solution. After addition of ISO, the Ca^2+^ transient amplitude reached steady state after ~1 min. The cardiomyocytes were stimulated for another 30 s before measurements were made of the Ca^2+^ transients. Ca^2+^ waves were measured in a 10-s pause after the stimulation period. SR Ca^2+^ removal rate was estimated as the average rate of decay (*k* = 1/tau) that was taken from the last three Ca^2+^ transients before a pause. SR Ca^2+^ content was measured as peak fluorescence after rapid application of 10 mM caffeine at 1 Hz in the presence and absence of ISO. SERCA2-dependent Ca^2+^ removal was measured as the difference between the decay rate of Ca^2+^ transients and the decay rate of SR Ca^2+^ release after exposure to 10 mM caffeine. Ca^2+^ removal rate by the sodium–calcium exchanger (NCX) and plasma membrane Ca^2+^ ATPase (PMCA) was measured as the decay rate of the caffeine-induced Ca^2+^ release.

The relative increase in diastolic Ca^2+^ during pacing was measured as the difference in mean F_0_ between 1, 2, and 4 Hz for three Ca^2+^ transients after 30 s stimulation.

Fractional release was measured as mean F of 3 Ca^2+^ transients after 30 s stimulation divided with F for caffeine release.

Confocal microscopy line-scan imaging was performed using a Zeiss LSM 7 Live confocal microscope (Carl Zeiss Microscopy, LCC, NY, USA). Cardiomyocytes were stimulated at 1 and 4 Hz in a protocol that was similar to the one described for whole-cell Ca^2+^ imaging measurements of Ca^2+^ waves, but with a 6-s post-stimulation rest period in which Ca^2+^ sparks were recorded.

### Beta-Adrenoceptor Quantification by Radioligand Binding

To assess beta-adrenoceptor density, a radioligand binding assay was performed on left ventricles snap frozen in liquid nitrogen. Crude cell membrane fractions were prepared as described by Krobert et al. (23). Radioligand binding was performed as described by Ramberg et al. (24), where membranes were incubated with [^125^I]-(–)iodocyanopincolol (0.066 nM) and the indicated concentration of either CGP20712A or ICI118551 for 2 h at 23°C. Data were fitted to a two-site binding model, and high and low binding affinities (*p*K_i_) were calculated in GraphPad Prism 8.0.1 using a K_d_ of 0.04 nM (affinity of [^125^I]-(–)iodocyanopincolol was determined in the left ventricular membranes). Beta_1_-adrenoceptor density was determined as an average of high-affinity CGP20712A and low-affinity ICI118551 binding in the same heart. Similarly, beta_2_-adrenoceptor density was determined as an average of high-affinity ICI118551 and low-affinity CGP20712A binding in the same heart.

### Phosphoprotein Analysis

Western blots were performed on total protein homogenates from the left ventricles that had been stored at −70°C. Homogenates were denatured at 100°C for 5 min or at 37°C for 10 min in a sample buffer that contained 50% sucrose, 7.5% sodium dodecyl sulfate (SDS), 62.5 mM Tris–HCl, 2 mM ethylenediaminetetraacetic acid (EDTA), 0.2 M dithiothreitol (DTT), and 0.01% bromophenol blue. Proteins were then fractionated according to size on 4–15% Criterion TGX gels (26 wells, 15 μl, Cat no. 567-1085, Bio-Rad Laboratories, Oslo, Norway) and blotted on 0.45 μM polyvinylidene fluoride (PVDF) membranes (GE Healthcare, Oslo, Norway). The examination of phospholamban (Plb) was performed through use of 18% Criterion TGX gels (26 wells, 15 μl, Cat no. 567-1075, Bio-Rad Laboratories, Oslo, Norway). Membranes were blocked in 5% non-fat milk in Tris-buffered saline with 0.1% polysorbate 20 for 1 h at room temperature. Then, they were incubated with primary antibody overnight at 4°C and with secondary antibody for 1 h at room temperature. Blots were developed by application of enhanced chemiluminescence (ECL prime, GE Healthcare, Oslo, Norway), and signals were quantified using ImageQuant software (GE Healthcare, Oslo, Norway).

The primary antibodies for protein detection were anti-RYR2 phosphoserine-2814 (A010-31, Badrilla, Leeds, UK), anti-RYR2 phosphoserine-2808 (A010-30, Badrilla, Leeds, UK), ryanodine receptor (MA3-916, Thermo Fisher Scientific Inc., Rockford, IL, USA), anti-CaMKII (phospho-threonine286) (ab32678, Abcam PLC, Cambridge, UK), CaMKIIδ (22), anti-phospholamban phosphoserine-16, (A010-12, Badrilla, Leeds, UK), anti-phospholamban phospho-threonine-17, (A010-13, Badrilla, Leeds, UK), and anti-SERCA2a, Cat no. MA3-919 (Thermo Fisher Scientific Inc., Rockford, IL, USA), beta_1_ adrenergic receptor antibody (PA1-049, Thermo Fisher), and monoclonal antivinculin antibody produced in mouse (V9131, Sigma-Aldrich). Secondary antibodies were antirabbit or antimouse immunoglobulin G (IgG) horseradish peroxidase (HRP)-linked whole antibodies, Cat no. NA934/NA931 (GE Healthcare, Oslo, Norway), diluted in the ratio 1:5,000. Glyceraldehyde 3-phosphate dehydrogenase (GAPDH) (v-18, sc-20357, Santa Cruz Biotechnology Inc., CA, USA) was used as the loading control for all Western blots, except for phosholamban, and for beta_1_ adrenergic receptor, for which vinculin was used. The membrane was reprobed with the specified antibodies with stripping of the membrane between each antibody when GAPDH was used as the loading control. HeLa Whole-cell lysate (sc-2200, Santa-Cruz) was used as a positive control for Western blots of beta_1_-adrenoceptor.

### Statistical Analysis

All experiments and analyses were performed by investigators who were blinded to the phenotype and group identity of each cell and animal. Statistical tests were selected based on advice from an external statistical advisor. A paired Student's *t*-test was used to compare the effect of exercise training on VO_2max_ at baseline and after 5 weeks within each group. Unpaired nested ANOVA was used to compare ET and SED for all results from whole-cell Ca^2+^ imaging. In addition, two-way ANOVA was used to compare difference between ET and SED in the presence or absence of ISO at all frequencies for Ca^2+^ waves, Ca^2+^ transient amplitude, and Ca^2+^ decay rate. A unpaird Student's t-test was used to compare differences in Ca^2+^ waves frequency across frequencies and in absense and presence of ISO for HF cardiomyocytes. Ca^2+^ spark frequency was analyzed by application of a Poisson test to adjust for skewed distribution. One-way ANOVA was used when comparing beta-adrenoceptors. Bonferroni, Holm–Sidak, or Tukey's correction was performed when appropriate. Normal distribution was assessed with Shapiro–Wilk test. All statistics were performed by using IBM SPSS statistics 27, Sigmaplot 12.5 or R software (version 3.0.2, The R Foundation for Statistical Computing). *P* < 0.05 was considered statistically significant. Results are reported as the mean ± standard error of mean (SEM).

## Results

### Heart Failure Model, Exercise Training, and Aerobic Capacity

**Figure 1:**
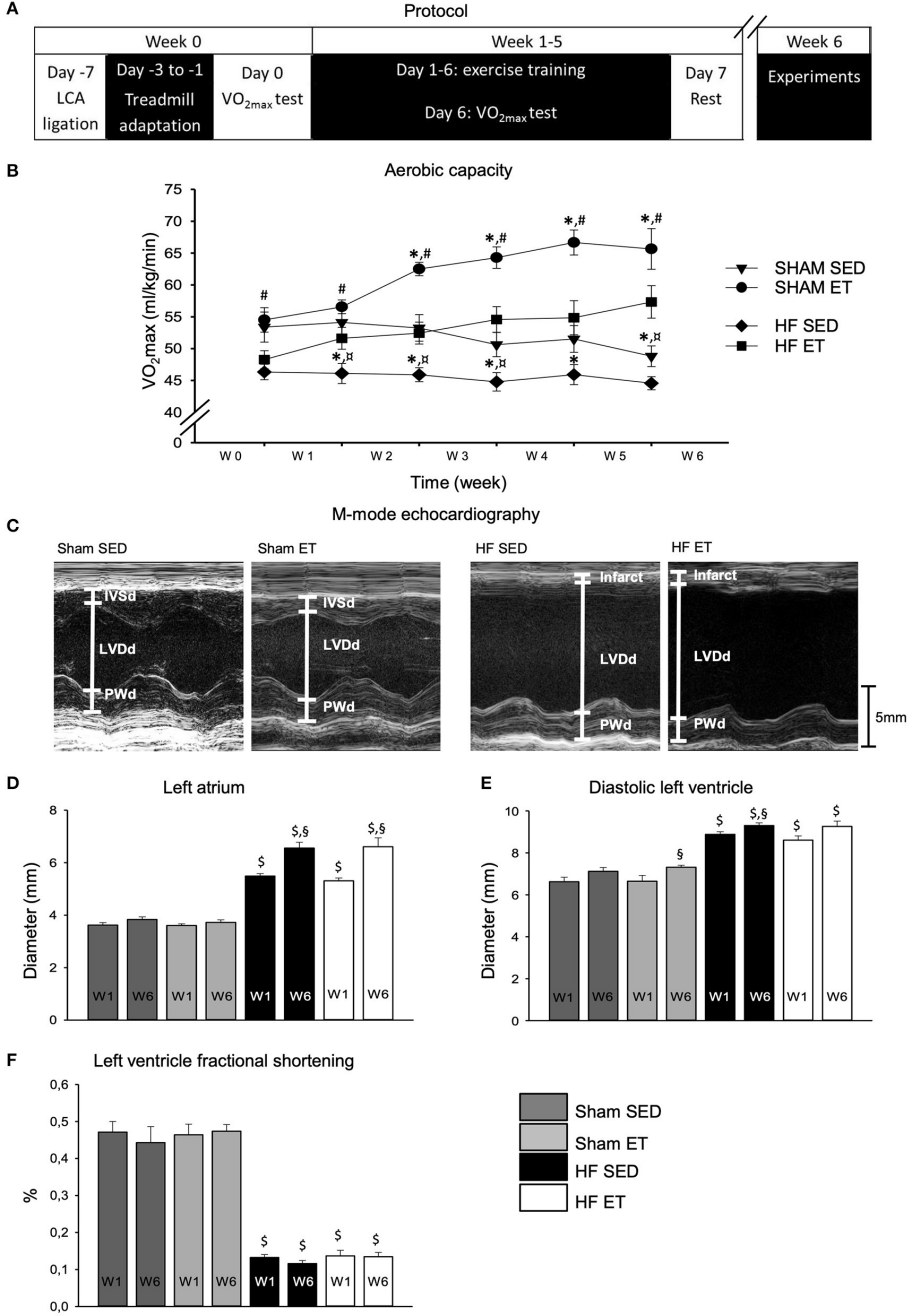
Aerobic capacity and echocardiographic parameters. High-intensity exercise training improved aerobic capacity in sham-ET and HF-ET rats. **(A)** Exercise training protocol. **(B)** Mean VO_2max_ measurements from sham and heart failure (HF) rats. Number of rats in each group (SED/ET): sham 15/14, HF 13/10. Echocardiographic parameters were used for stratification of rats to the sham and HF groups. Representative M-mode images from the left ventricle in the parasternal long axis are shown for sham-SED **(C)** (left, n = 9), sham-ET (middle left, n = 8), HF-SED (middle right, n = 12), and HF-ET rats (left, n = 8). Bar graphs of the **(D)** left atrium diameter, **(E)** left ventricular diameter in diastole, and **(F)** left ventricle fractional shortening represent mean ± SEM. *P* < 0.05: ^$^sham vs. HF; ^*^SED vs. ET; ^#^HF-ET vs. sham-ET; ^§^week 1 vs. week 6; ¤HF SED vs. sham SED (Paired Student's *t*-test).

We performed left coronary artery ligation on 23 male Wistar rats to induce large left ventricular myocardial infarctions. Sham operations were performed on 29 rats. One week after surgery, all rats were examined by echocardiography before randomization to ET or standard sedentary conditions (SED) (Figure 1A).

One week after surgery, the aerobic capacity of HF-ET rats was 86% of that of the sham-ET rats (*P* < 0.05) but did not differ from that of the HF-SED rats (Figure 1B). During the ET protocol, VO_2max_ increased in both ET groups, as expected. After 5 weeks of ET, VO_2max_ in the HF-ET group was 127% of that of the HF-SED group (*P* < 0.05), while VO_2max_ in the sham-ET group was 119% of that of the sham-SED group (*P* < 0.05, Figure 1B). However, VO_2max_ in the HF-ET group was only 88% of that of the sham-ET group (Figure 1B, *P* < 0.05).

Echocardiography was performed before randomization and repeated after completion of the ET protocol (Figure 1C). As per definition, the left atrial diameter was larger in the HF groups than in the sham groups at the time of randomization 1 week after surgery and had increased further in both HF groups when examined at week 6 (*P* < 0.05, Figure 1D). One week after surgery, HF rats also showed a clear phenotype with increased left ventricular diameter and reduced fractional shortening (*P* < 0.05, Figures 1E,F). During 5 weeks of ET, the left ventricular diastolic diameter (Figure 1E) and fractional shortening remained unchanged in the HF-ET group (Figure 1F), and ET did not affect any of the echocardiographic parameters in sham-operated rats (Figures 1D–F).

### Spontaneous Calcium Release in Cardiomyocytes From HF Rats

**Figure 2:**
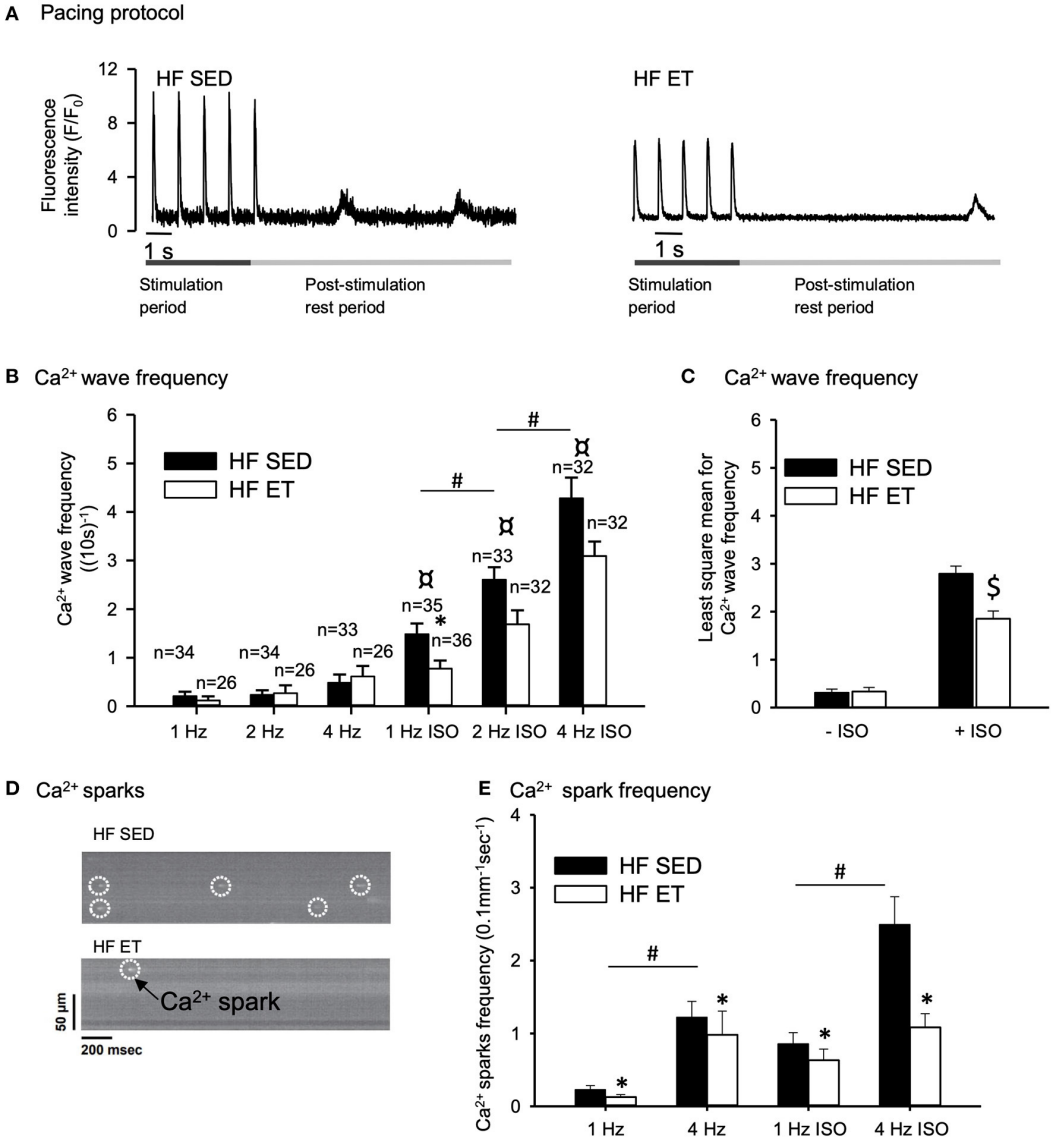
Effect of exercise training on spontaneous Ca^2+^ release in heart failure (HF) cardiomyocytes. **(A)** Representative Ca^2+^ wave recordings and **(D)** confocal images of Ca^2+^ sparks are shown after field stimulation of HF-SED and HF-ET cardiomyocytes that were exposed to isoprenaline sulfate (ISO). Pacing at 1, 2, and 4 Hz was provided, and ISO was added to the experimental solution. Bar graphs of **(B)** Ca^2+^ wave frequency, **(C)** Ca^2+^ wave frequency across all pacing frequencies, and **(E)** Ca^2+^ spark frequency represent mean ± SEM. Number of rats in each group (SED/ET): Ca^2+^ wave frequency: *n* = 6/5, Ca^2+^ spark frequency: *n* = 10/8. Number of cells used for measurements of Ca^2+^ spark frequency (SED/ET): without ISO 101/81, with ISO 88/75. *P* < 0.05: ^*^HF-SED vs. HF-ET (unpaired nested ANOVA); ^*^(Poisson test for Ca^2+^ spark frequency, Bonferroni); ^#^1 Hz vs. 2 and 4 Hz (Unpaired Student's *t*-test); ¤ +ISO vs. –ISO at same frequency (Unpaired Student's *t*-test), ^$^least square mean for HF-SED vs. HF-ET (two-way ANOVA, Holm-Sidak).

We isolated cardiomyocytes from the left ventricles of the HF-ET and HF-SED rats at week 6 for the analysis of RyR2-dependent spontaneous Ca^2+^ release. We first recorded cardiomyocyte-wide propagated Ca^2+^ release events, i.e. Ca^2+^ waves, after a train of electrical stimuli at increasing frequencies (Figures 2A,B). We performed this protocol in the absence and presence of ISO to simulate sympathetic activation with beta-adrenoceptor stimulation. Ca^2+^ wave frequency increased with increasing pacing frequency and during exposure to ISO (Figure 2B). Importantly, Ca^2+^ wave frequency during ISO was lower in HF-ET cardiomyocytes than in those of HF-SED rats at 1 Hz (Figure 2B) and when considered across all frequencies with two-way ANOVA (Figure 2C). This finding indicated that ET stabilized Ca^2+^ handling in HF.

We also measured local Ca^2+^ release events, i.e., Ca^2+^ sparks, by confocal microscopy after a train of electrical stimuli (Figure 2D). The frequency of Ca^2+^ sparks increased with stimulation frequency (from 1 to 4 Hz), both in the presence and absence of ISO (P < 0.05), and was clearly lower in the HF-ET than in the HF-SED cardiomyocytes (Figure 2E). This further indicated that ET stabilized SR Ca^2+^ handling in HF.

### Systolic Ca^2+^ Release in Cardiomyocytes From HF Rats

**Figure 3:**
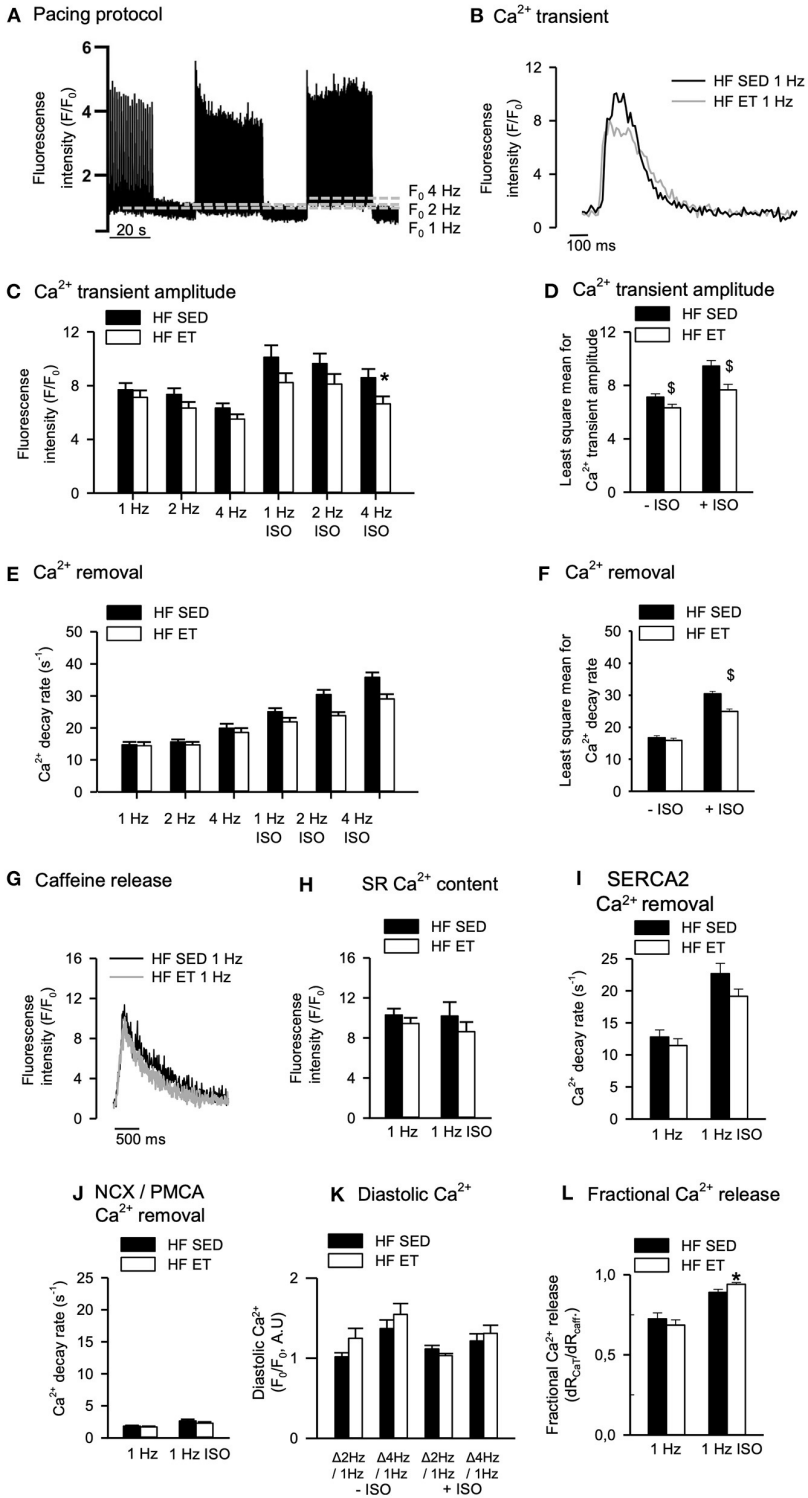
Effects of exercise training on Ca^2+^ handling in heart failure (HF) cardiomyocytes. **(A)** Representative pacing protocol and **(B)** Ca^2+^ transients for HF-SED and HF-ET cardiomyocytes during 1 Hz stimulation and exposure to isoprenaline sulfate (ISO). **(C)** Ca^2+^ transient amplitude was reduced after exercise training at high stimulation frequency and ISO exposure and **(D)** across all frequencies with and without ISO exposure. **(E,F)** Ca^2+^ decay rate was lower across all frequencies with ISO exposure. **(G)** Representative tracings of SR Ca^2+^ content, measured by peak F/F_0_ in response to a rapid application of 10 mM caffeine. Exercise training **(H)** did not affect SR Ca^2+^ content and **(I)** did not alter Ca^2+^ removal by SERCA2a or sodium–calcium exchanger (NCX) and plasma membrane Ca^2+^ ATPase (PMCA), as **(J)** measured by the decay rate of the caffeine-induced Ca^2+^ release. **(K)** Exercise training did not alter diastolic Ca^2+^; **(L)** however, fractional release was higher in HF-ET compared to HF-SED when exposed to ISO. Number of rats (SED/ET): 6/5. Number of cells in **C–F**, **K** (SED/ET): –ISO 34/26, +ISO 35/36. Number of cells in **H–J**, **L** (ET/SED): HF 26/22, ISO 22/28. Bar graphs represent mean ± SEM. *P* < 0.05: ^*^HF-SED vs. HF-ET (unpaired nested ANOVA), ^$^least square mean for HF-SED vs. HF-ET (two-way ANOVA, Holm-Sidak).

If ET is to be used as an antiarrhythmic intervention, negative effects on systolic Ca^2+^ release responsible for contraction should not outweigh the positive effects of ET on RyR2-dependent spontaneous Ca^2+^ release associated with arrhythmias. Surprisingly, however, when we analyzed Ca^2+^ transients across different pacing frequencies, the amplitude was lower in HF-ET than HF-SED both in the absence and presence of ISO (Figures 3A–D). Ca^2+^ transient decay rate was also lower in HF-ET than HF-SED, but only during exposure to ISO (Figures 3E,F). To explain these findings, SR Ca^2+^ content and Ca^2+^ removal were analyzed during caffeine exposure (Figure 3G). However, no difference in SR Ca^2+^ content was observed between HF-ET and HF-SED (Figure 3H) nor could the slowed removal be attributed to SERCA2-dependent or sarcolemmal Ca^2+^ removal alone (Figures 3I,J). We also analyzed the increase in diastolic Ca^2+^ that is expected in response to increased pacing frequencies but found no difference between HF-ET and HF-SED (Figure 3K). Lastly, we calculated fractional SR Ca^2+^ release but only observed a small increase in HF-ET compared HF-SED during ISO, which cannot explain stabilized RyR2-dependent SR Ca^2+^ release (Figure 3L) (25).

### Phosphoproteins in Left Ventricles From HF Rats

**Figure 4:**
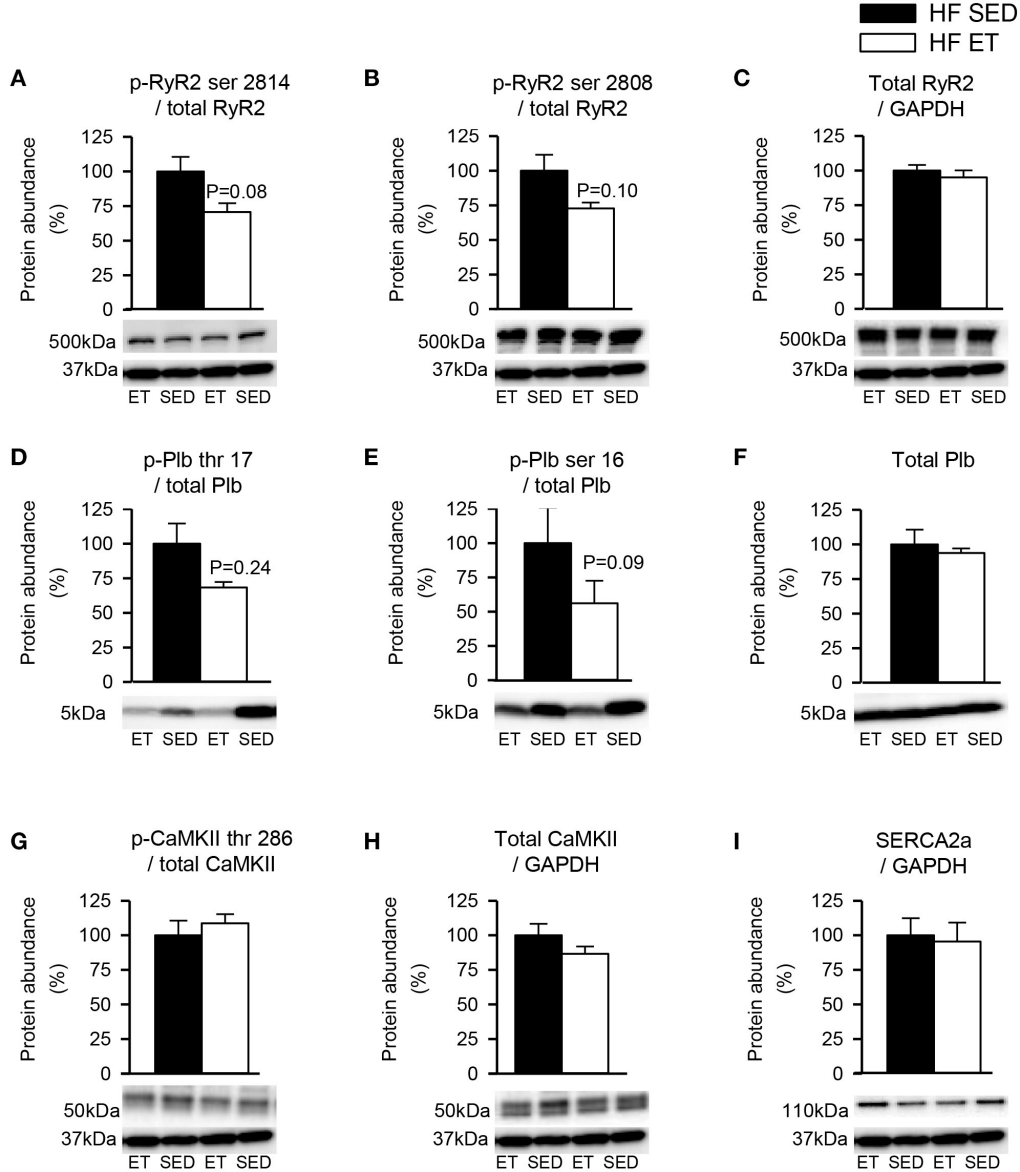
Protein phosphorylation levels in heart failure (HF) left ventricles. Exercise did not decrease phosphorylation at sites that are known to be involved in destabilization of RyR2. Phosphoprotein levels were normalized to total protein levels. RyR2 phosphorylated at **(A)** serine 2814 (p-RyR2 ser 2814) and **(B)** serine 2808 (p-RyR2 ser 2808) were normalized to **(C)** total RyR2. Plb phosphorylated at **(D)** threonine 17 (p-Plb thr 17) and **(E)** serine 16 (p-Plb ser 16) were normalized to **(F)** total Plb and CaMKII phosphorylated at **(G)** threonine 286 (p-CaMKII thr 286) was normalized to **(H)** total CaMKII. **(I)** SERCA2a level was unaltered by exercise training. Glyceraldehyde 3-phosphate dehydrogenase (GAPDH) was used as loading control, except for Plb, and the membrane was reprobed with the specified antibodies with stripping of the membrane between each antibody, shown at 37 kDa. **(C,F,H,I)** Total protein levels were normalized to GAPDH levels. Number of rats (SED/ET): 6/5.

To explore other potential explanations for the stabilization of RyR2-dependent spontaneous Ca^2+^ release that was observed after ET, phosphoproteins levels were quantified in HF-ET and HF-SED (Figure 4). We focused on RyR2 and the SERCA/Plb system as the proteins that are mainly responsible for SR Ca^2+^ release and reuptake. However, no differences in these proteins or their major regulatory phosphorylation sites were observed (Figures 4A–I). The Plb/SERCA2a ratio was also unaltered in HF-ET compared with that in HF-SED (*P* = 0.68).

### Effects of Exercise Training in Sham-Operated Rats

**Figure 5:**
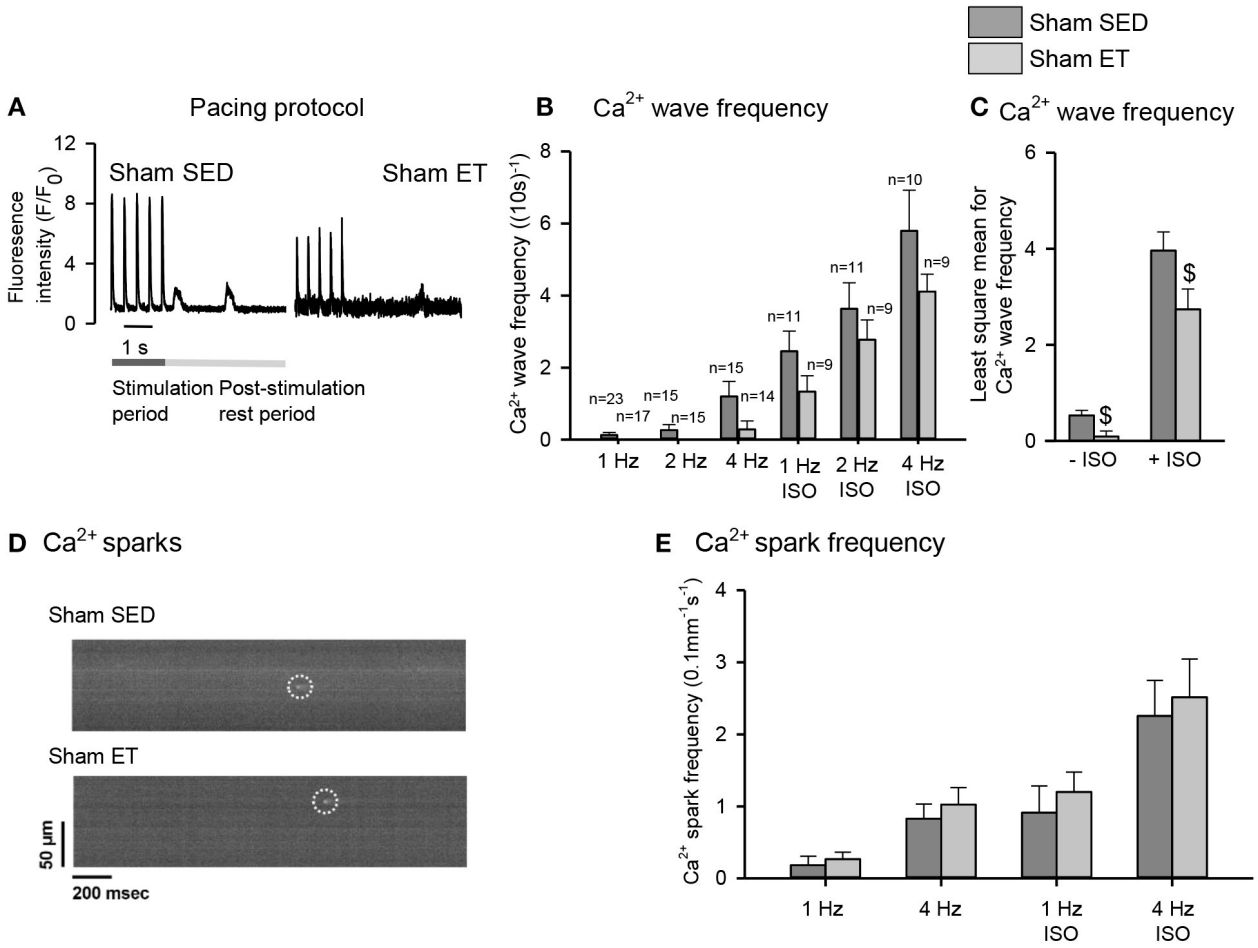
Effect of exercise training on spontaneous Ca^2+^ release in sham cardiomyocytes. **(A)** Representative Ca^2+^ wave recordings, bar graphs of **(B)** Ca^2+^ wave frequencies at different pacing frequencies in the absence and presence of isoprenaline sulfate (ISO) and **(C)** two-way ANOVA comparing HF-ET with HF-SED. **(D)** Confocal images of Ca^2+^ sparks, and **(E)** bar graphs of Ca^2+^ spark frequency after different pacing frequencies. Number of rats (SED/ET): Ca^2+^ waves 4/3; Ca^2+^ sparks 7/6; number of cells (SED/ET): Ca^2+^ sparks: –ISO (60/53), +ISO (46/48); Ca^2+^ handling: –ISO (23/17), +ISO (10/9). *P* < 0.05: ^$^least square mean for sham-SED vs. sham-ET (two-way ANOVA, Holm-Sidak).

**Figure 6:**
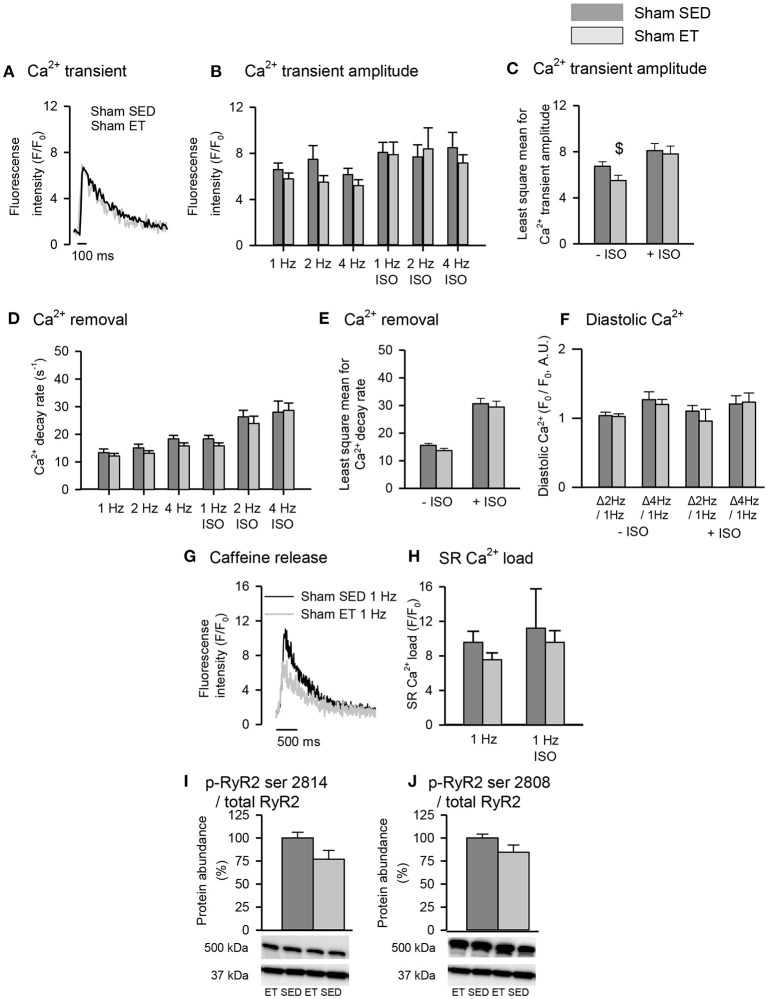
Effect of exercise training on Ca^2+^ handling in sham cardiomyocytes. **(A)** Representative Ca^2+^ transient recordings is shown after field-stimulation of sham-SED and sham-ET cardiomyocytes. Stimulation of 1, 2, and 4 Hz was provided, and isoprenaline sulfate (ISO) was added to the experimental solution. Bar graphs of **(B,C)** Ca^2+^ transient amplitude, **(D,E)** Ca^2+^ removal and **(F)** diastolic Ca^2+^ represent mean ± SEM. **(G)** Representative tracings of SR Ca^2+^ content, measured by peak F/F_0_ in response to a rapid application of 10 mM caffeine at 1 Hz. **(H)** Bar graphs of SR Ca^2+^ load, represents mean ± SEM. Phosphoprotein levels were normalized to total protein levels. RyR2 phosphorylated at **(I)** serine 2814 (p-RyR2 ser 2814) and **(J)** serine 2808 (p-RyR2 ser 2808). **(I,J)** Glyceraldehyde 3-phosphate dehydrogenase (GAPDH) was used as loading control, and the membrane was reprobed with the specified antibodies with stripping of the membrane between each antibody, shown at 37 kDa. Number of rats (SED/ET): Ca^2+^ handling (4/3); proteins (5/5). Number of cells (SED/ET): Ca^2+^ handling: –ISO (23/17), +ISO (10/9). *P* < 0.05: ^$^least square mean for sham-SED vs. sham-ET (two-way ANOVA, Holm-Sidak).

To control for untoward effects of ET, we performed most experiments in parallel in sham-ET and sham-SED rats (Figures 5, 6). The ET protocol lowered Ca^2+^ wave frequency even in sham-ET compared to sham-SED both in the presence and absence of ISO when compared across all frequencies with two-way ANOVA (Figures 5A–C). However, Ca^2+^ spark frequency was similar in sham-ET and sham-SED (Figures 5D,E). ET had no effect on Ca^2+^ transients amplitude (Figures 6A–C), Ca^2+^ removal rate, diastolic Ca^2+^ (Figures 6D–F), SR Ca^2+^ content (Figures 6G,H), or RyR2 phosphorylation (Figures 6I,J) in sham-operated animals.

### Effects of Exercise Training on Beta-Adrenoceptors in HF and Sham-Operated Rats

**Figure 7:**
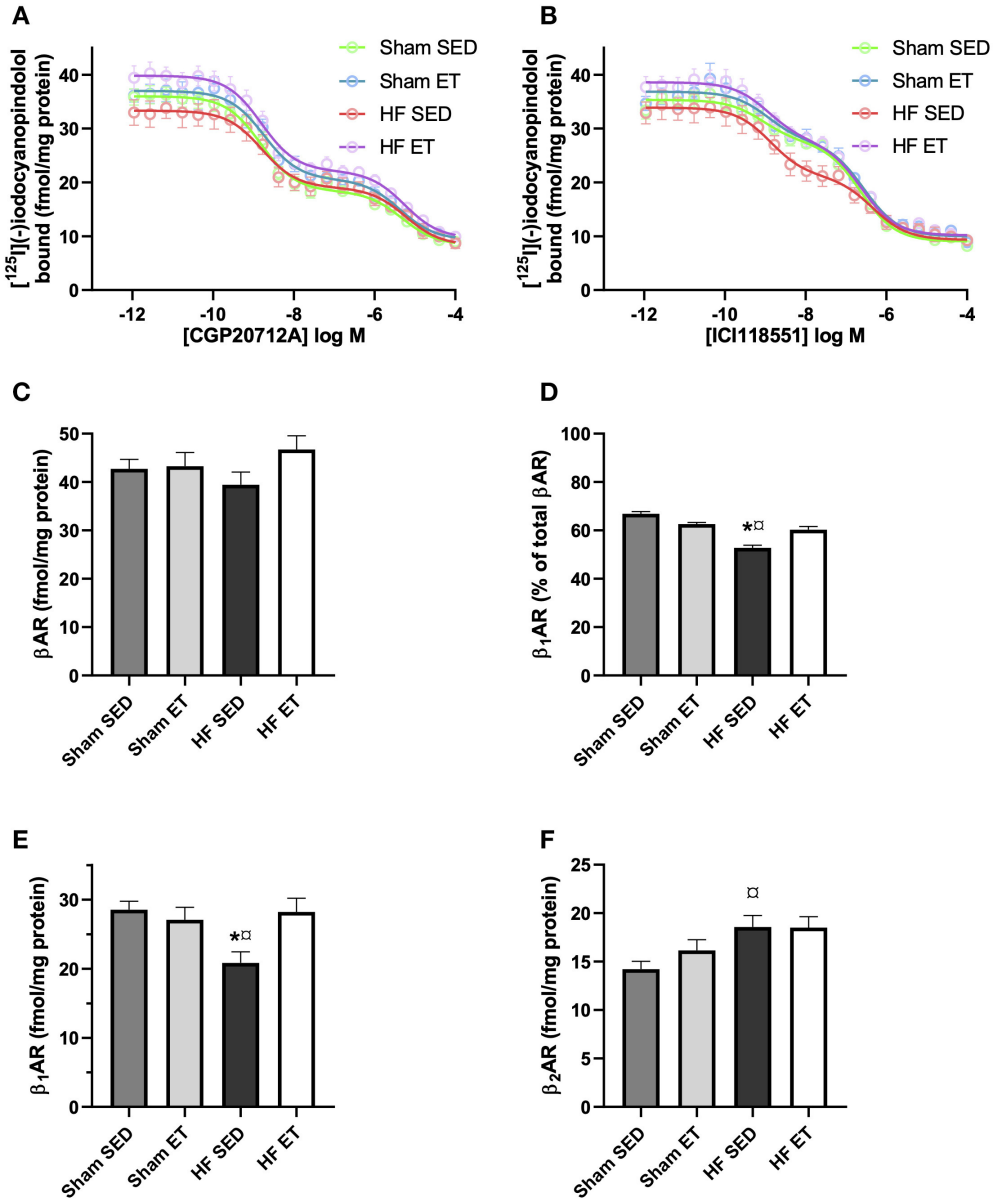
Beta-adrenoceptor density in heart failure (HF) and sham left ventricles. Competitive binding assays between [^125^I]-(–)iodocyanopindolol and the **(A)** beta_1_-adrenoceptor selective ligand CGP20712A or the **(B)** beta_2_-adrenoceptor selective ligand ICI118551 in membranes from the left ventricle from animals with the indicated treatment. The **(C)** total beta-adrenoceptor density and **(D)** percentage of beta_1_-adrenoceptor was determined by a two-site binding model, where the average of high-affinity CGP20712A and low-affinity of ICI118551 is presented. **(E)** Beta_1_-adrenoceptor density was determined as the average of high affinity of CGP20712ACFP20712A displacement and low affinity of ICI118551 displacement. **(F)** Beta_2_-adrenoceptor density was determined as the average of high affinity of ICI118551 displacement and low affinity of CGP20712ACFP20712A displacement. The data shown are mean ± SEM of five (sham SED), four (sham ET), six (HF SED), and six (HF ET) animals. *P* < 0.05: ^*^SED vs. ET, ^¤^HF SED vs. sham SED (one-way ANOVA, Tukey's correction).

As an alternative explanation for our observations, we examined if beta-adrenoceptor density was affected by exercise. For this, we used radioligand binding on membranes from left ventricular cardiomyocytes. The beta_1_-adrenoceptor selective ligand CGP20712A displayed a *p*K_i−high_ of 9.21 ± 0.02 and a *p*K_i−low_ of 5.7 ± 0.03 (Figure 7A), while the beta_2_-adrenoceptor selective ligand ICI118551 displayed a *p*K_i−high_ of 9.32 ± 0.04 and a *p*K_i−low_ of 6.9 ± 0.04 (Figure 7B), similar to previously reported values (26). The total beta-adrenoceptor density did not differ between the four groups (HF-ET, HF-SED, sham-ET, sham-SED, Figure 7C). Importantly, however, the beta_1_-adrenoceptor density was significantly reduced in the HF-SED compared to sham-SED but increased back to normal levels in HF-ET (Figures 7D,E). The beta_2_-adrenoceptor density, on the other hand, was increased in HF-SED compared to sham-SED and was not affected by ET (Figure 7F). In contrast to the effects of ET on membrane beta_1_-adrenoceptor density, total abundance of the receptor measured by Western blotting was not altered by ET (Supplementary Figure 1).

## Discussion

We tested the hypothesis that ET could stabilize RyR2-dependent SR Ca^2+^ release associated with arrhythmias in post-MI HF rats. We subjected rats with HF to a 5-week ET protocol that was initiated 1 week after the induction of MI by ligation of the left coronary artery. This protocol increased the aerobic capacity of HF-ET rats to 127% of that of HF-SED rats but did not prevent a further increase in atrial diameter and had no impact on left ventricular diameter or on contractile function measured as fractional shortening. ET reduced the frequency of spontaneous Ca^2+^ release events in left ventricular cardiomyocytes, as indicated by a reduced frequency of Ca^2+^ waves and Ca^2+^ sparks in HF-ET cells compared with the HF-SED cells. In situations associated with increased adrenergic stress and beta-adrenoceptor stimulation, the HF-ET group exhibited lower Ca^2+^ transient amplitudes and decay rates than the HF-SED group, but no significant changes in RyR2, SERCA2 or their major regulatory proteins or phosphorylation sites. Sham-operated ET rats also exhibited a 135% increase in aerobic capacity compared to sham-SED and lower Ca^2+^ wave frequency when measured across all frequencies. However, we observed no changes in echocardiographic parameters or changes in Ca^2+^ measurements or SR Ca^2+^ regulatory proteins that can explain this. Since the Ca^2+^ regulatory proteins could not explain our main observation, we tested an alternative hypothesis and found that beta_1_-adrenoceptors were downregulated in HF-SED but normalized with ET.

### Rat Model of Post-MI HF and RyR2 Dysfunction in Perspective

The pathophysiological changes that occur in post-MI HF are highly complex and progress over time (27). After a large MI, structural and mechanical alterations occur at the organ level, as well as in cardiomyocytes and the extracellular matrix (28). The progression of these alterations leads to different mechanisms being responsible for increased risk of arrhythmias at different time points after an MI (29). Altered Ca^2+^ handling due to perturbed expression and function of Ca^2+^ handling proteins is involved in arrhythmogenesis at all stages after MI, mainly because these alterations increase the risk of triggered activity (30, 31). Dysfunctional RyR2 is key in this arrhythmia mechanism and has been observed in several animal models of established post-MI HF (32–34). Our model is in line with other models of HF, with reduced aerobic capacity, severely dilated left ventricular diameters, and increased left-atrial diameters as a sign of long-standing increase in end-diastolic pressure, as well as reduced density of beta_1_-adrenoceptors (35, 36). Many models of HF with a similar phenotype show post-translational modifications of RyR2, especially increased phosphorylation (12). Thus, dysfunction and modification of RyR2 have received much attention as potential targets for prevention of arrhythmias in HF (37). Our model is therefore highly relevant to test the hypothesis that ET can stabilize RyR2.

### Exercise Training as Antiarrhythmic Therapy in Post-MI HF

There is widespread interest in HF pathophysiology, RyR2 dysfunction, and the beneficial effects of ET in HF. Therefore, it is a surprise that data on the effects of ET on RyR2 function in post-MI HF are scarce. Bonilla et al. subjected dogs with anterior MI to 10 weeks of ET and showed a highly reduced tendency toward ventricular fibrillation compared with sedentary dogs (38). In line with our study, they showed that the frequency of Ca^2+^ sparks in ventricular cardiomyocytes was reduced in dogs in the exercise group and that the abundance of RyR2 that was phosphorylated at serine 2814 was also reduced, compared with the sedentary group. However, there was no data that indicated that the dogs had HF. Kemi et al. performed a study of post-MI HF rats that showed many similarities with our study, although they employed a different rat strain (39). They too found a positive effect on Ca^2+^ wave frequency, but they did not analyze phosphoproteins. In other publications, the same group has provided compelling evidence that ET can have beneficial effects on Ca^2+^ handling and on T-tubule structure in normal rats (5, 17, 20, 40). Based on these and other data of disease models that show dysfunctional RyR2 (17, 18), we hypothesized that ET could have beneficial effects on RyR2 function. We focused specifically on serine 2808 and 2814 in RyR2, as there is mounting evidence that phosphorylation of these residues contributes to SR Ca^2+^ leak in HF and that prevention of such phosphorylation can prevent arrhythmogenic Ca^2+^ release (41, 42). It is therefore interesting that we observed beneficial effects on spontaneous SR Ca^2+^ release, without significant alterations in the abundance of key SR Ca^2+^ handling proteins, or CaMKII-or protein kinase A (PKA)-dependent phosphorylation. Although previous studies have clearly shown that ET can exert beneficial effects on SR Ca^2+^ handling through attenuated phosphorylation by CaMKII especially, our results show that other mechanisms may also contribute to a similar result. Interestingly, we observed that Ca^2+^ transient amplitude and reuptake of Ca^2+^ was lower in HF-ET rats during adrenergic stress. It was therefore somewhat surprising that this coincided with increased beta_1_-adrenoceptor density compared to HF-SED. Our radioligand binding experiments show that the number of beta_1_-adrenoceptors was reduced in HF rats and increased to normal levels by ET (Figure 7). Our experiments do not provide an explanation for how this is compatible with reduced reuptake or why the increased density was not reflected in the total abundance of the receptors when measured by Western blotting. We can only speculate that ET altered the relative pools of these receptors at the cell surface and receptors associated with the SR. Recent studies have shown that internalized and SR-bound beta_1_-adrenoceptors exert an important regulatory role in SR Ca^2+^ release (43). These possible effects of ET should be explored further in future studies.

### Limitations

We investigated Ca^2+^ handling after MI and ET and focused specifically on SR Ca^2+^ handling. Our studies lead us toward a role of beta-adrenoceptors in the effects of ET in HF rats, although the focus of the study did not allow us to conclude if this represents an explanation to reduced frequency of spontaneous Ca^2+^ release events after ET. Still, there is a risk that we missed a difference in phosphoprotein abundance after ET, due to low sample size (type II error) in our analysis, and such effects should therefore not be excluded. The reader should be aware that a one-sided test of CaMKII-dependent phosphorylation of RyR2 would have resulted in a *P* < 0.05. Based on previous data, the *a priori* assumption that ET would decrease (not increase) CaMKII activity could be claimed, thus supporting a one-sided test. Furthermore, our analyses of phosphoproteins were only performed in the absence of sympathetic stimulation. Analysis performed after f.ex. exposure to ISO could potentially have clarified some of the non-significant observations.

Another potentially contributing factor that should be included in future studies is altered cell ultrastructure. ET can effect cell size and has been shown to alter t-tubule organization (40). These aspects could be of importance but were not included in our study.

Our study is limited to cellular phenomena and does not include measurements of *in vivo* arrhythmias. This limits the potential for extrapolation to propensity for arrhythmias *in vivo*. However, other mechanisms are also important for antiarrhythmic effects of ET, such as alterations in repolarization reserve (38) or reductions in fibrosis levels (44), although the latter is debated after findings in healthy rats (45). As these other mechanisms were not the focus of our study, we would not have been able to ascertain a causal link between the cellular phenomena and arrhythmias regardless of any observed effects on arrhythmias.

Our model of HF is based on induction of large MIs, which leads to clear mechanical and structural alterations in the left ventricle at an early time point after the MI. Any extrapolation to HF that develops after smaller MIs, which involves less remodeling, is unclear. Especially, we did not separate cardiomyocytes according to relative localization or distance from the MI, although the effects of ET could vary between regions (46, 47).

We chose to start high-intensity ET at an early time point after MI. High-intensity ET was chosen based on previous studies in rats (6). Moderate-intensity ET might have been more in line with what patients can perform, although high-intensity interval training is feasible even in patients with severe HF (48). Any extrapolation to correspondingly large MIs in humans is speculative, and no conclusion regarding the time at which ET should be initiated in patients can be drawn from studies in rats. The early initiation that was chosen in our study could mean that remodeling processes were still highly active, which could have affected our results. However, it could be argued that this is a reason to stress early initiation of ET in order to affect beneficially the remodeling process. We did not compare effects of exercise in HF and sham rats for all data, as this was not the focus of our study. The effects of ET could be different in diseased and normal animals, and the therapeutic potential should be tested in relevant disease models.

## Conclusions

ET can stabilize RyR2-dependent Ca^2+^ release in post-MI HF. Our data indicate that the protective mechanism involves regulation of both SR Ca^2+^ release and reuptake, and that effects of ET at several levels of the sympathetic signaling pathways controlling SR Ca^2+^ release should be explored in future studies.

## Data Availability Statement

The raw data supporting the conclusions of this article will be made available by the authors, without undue reservation.

## Ethics Statement

The animal study was reviewed and approved by Norwegian National Committee for Animal Welfare.

## Author Contributions

TD, MS, RM, JA, MF, MH, KA, KH, and IS conceived and designed the analysis, collected data, contributed to data analysis, performed the analysis, and wrote the paper. FL, WL, OS, and MS conceived and designed the analysis, contributed to data analysis, and wrote the paper. All authors contributed to the article and approved the submitted version.

## Conflict of Interest

The authors declare that the research was conducted in the absence of any commercial or financial relationships that could be construed as a potential conflict of interest.
