# Supplementary figures and images for: Exercise Training Stabilizes RyR2-Dependent Ca2+ Release in Post-infarction Heart Failure
Source: Front Cardiovasc Med. 2021 Jan 25;7:623922. doi: 10.3389/fcvm.2020.623922 (PMC7868397; doi:10.3389/fcvm.2020.623922)

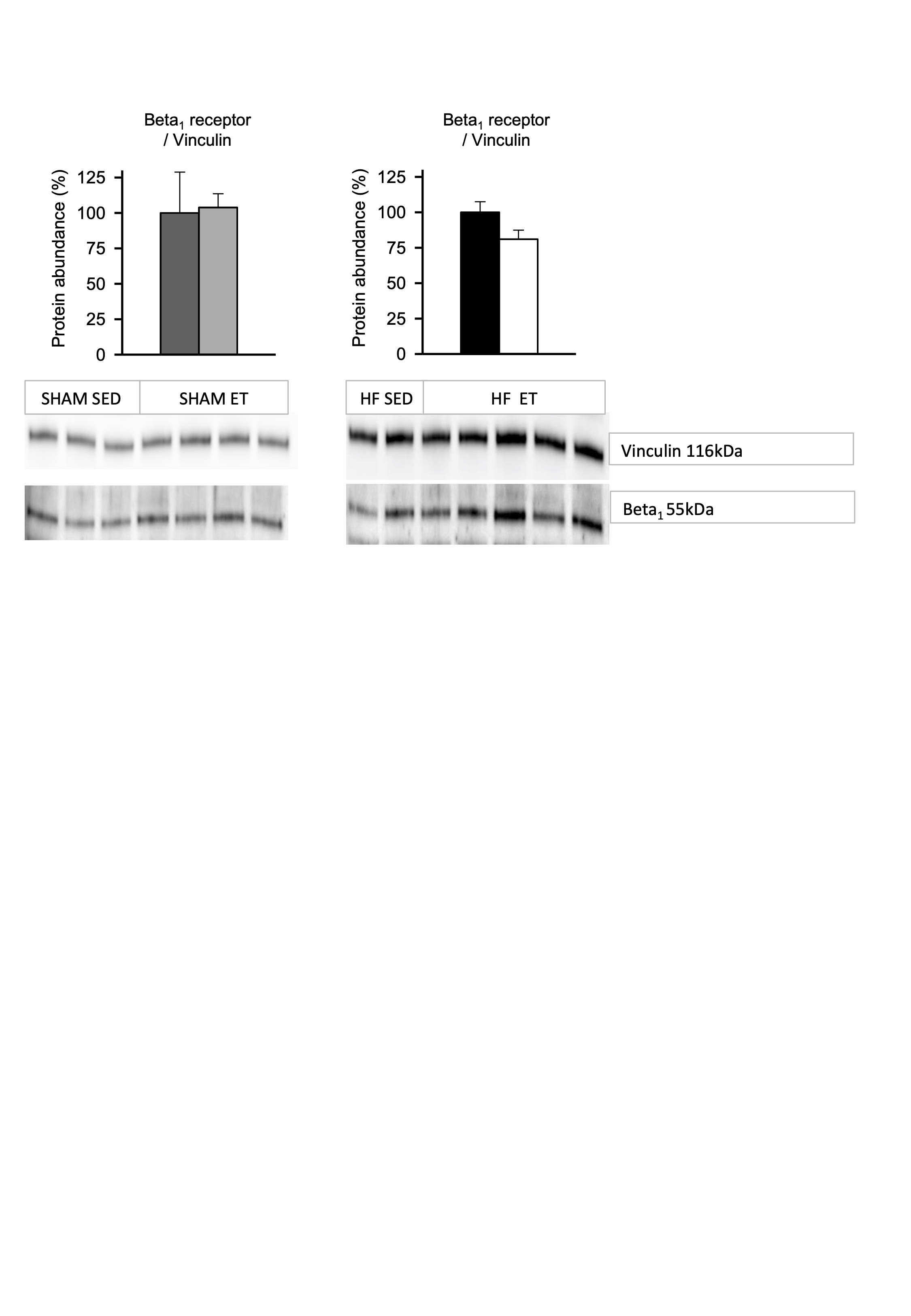

Supplement: Supplementary file 1 [file Image_1.JPEG]
